# New approach of prediction of recurrence in thyroid cancer patients using machine learning

**DOI:** 10.1097/MD.0000000000027493

**Published:** 2021-10-22

**Authors:** Soo Young Kim, Young-Il Kim, Hee Jun Kim, Hojin Chang, Seok-Mo Kim, Yong Sang Lee, Soon-Sun Kwon, Hyunjung Shin, Hang-Seok Chang, Cheong Soo Park

**Affiliations:** a Department of Surgery, Ajou University College of Medicine, Suwon, Korea; b GN Systems Inc., Seoul, Korea; c Department of Surgery, CHA Ilsan Medical Center, Goyang-si, Korea; d Department of Surgery, Thyroid Cancer Center, Gangnam Severance Hospital, Institute of Refractory Thyroid Cancer, Yonsei University College of Medicine, Seoul, Korea; e Department of Mathematics/AI & Data Science, Ajou University, Suwon, Korea; f Department of Industrial Engineering, Ajou University, Suwon, Korea.

**Keywords:** inductive logic programming, machine learning, recurrence prediction, thyroid cancer, thyroid cancer recurrence

## Abstract

Although papillary thyroid cancers are known to have a relatively low risk of recurrence, several factors are associated with a higher risk of recurrence, such as extrathyroidal extension, nodal metastasis, and BRAF gene mutation. However, predicting disease recurrence and prognosis in patients undergoing thyroidectomy is clinically difficult. To detect new algorithms that predict recurrence, inductive logic programming was used in this study.

A total of 785 thyroid cancer patients who underwent bilateral total thyroidectomy and were treated with radioiodine were selected for our study. Of those, 624 (79.5%) cases were used to create algorithms that would detect recurrence. Furthermore, 161 (20.5%) cases were analyzed to validate the created rules. DELMIA Process Rules Discovery was used to conduct the analysis.

Of the 624 cases, 43 (6.9%) cases experienced recurrence. Three rules that could predict recurrence were identified, with postoperative thyroglobulin level being the most powerful variable that correlated with recurrence. The rules identified in our study, when applied to the 161 cases for validation, were able to predict 71.4% (10 of 14) of the recurrences.

Our study highlights that inductive logic programming could have a useful application in predicting recurrence among thyroid patients.

## 1 Introduction

Well-differentiated thyroid cancer (WDTC) is 1 of the most common types of endocrine malignancy comprising over 90% of all thyroid cancers. Furthermore, it has shown steadily increasing incidence over the last 3 decades.^[1,2]^ Currently, WDTC is the most prevalent cancer in Korea.^[2,3]^ Nonetheless, despite its increasing incidence, the thyroid cancer-related mortality rate remains low.^[4]^

The overall 5-year survival of WDTC is high at 97.9%, and that of low risk patients in stages I and II nearly at 100%.^[5]^ Interestingly, WDTC is unique in that it frequently metastasizes to the lymph nodes. Among the most frequent sites to which it metastasizes are the central lymph nodes. However, a metastasis to the central lymph node has only marginal effects on the long-term survival of patients.^[6–8]^

Although the mortality rate for thyroid cancer is low and 5-year survival rates are high, postoperative recurrence is the primary cause of death in thyroid cancer patients. Reoperations for recurrent thyroid cancer can cause serious complications in the patient's physical and mental health. In addition to accurate preoperative assessment and proper treatment, accurate risk stratification with close-follow-up to reduce recurrence and detect recurrence early are necessary.

While WDTC may remain indolent, recurrence rates are reported to be between 12 and 20%,^[9,10]^ with males showing higher recurrence rates than that of females. Larger tumor diameter, lymph node metastasis, and pathological tumor types have been reported to have a higher recurrence rate.^[9]^ The 2015 American Thyroid Associated guideline describes several factors that affect the risk of recurrence, such as extrathyroidal extension, lymph node involvement, multifocality, and BRAF gene mutation status.^[11]^ However, predicting disease recurrence and prognosis in patients undergoing thyroidectomy is clinically difficult.

Inductive logic programming (ILP) is a computer programming technique that is particularly helpful in aiding researchers with data mining and the knowledge discovery process.^[12]^ It has evolved from previous research on machine learning, logic programming, and inductive program synthesis.^[13]^ The objective of ILP is to discover a set of if-then rules that predicts the presence or absence of a disease or outcome. To generate rules, the following parameters are necessary: positive and negative examples; background knowledge about given examples; and user-defined constraints about what type of rules may be learned.^[12]^

Briefly, ILP is performed on the basis of the following given information:

- A background knowledge *B* represents the knowledge available before learning
- A set of positive examples *E*^+^ and a set of negative examples *E*^−^

The goal is to find hypotheses *H* (set of rules), where:

- All or almost all positive examples *e* ∊ *E*^+^ are covered by *H*
- No or few negative examples are covered by *H*.^[14]^

The advantages of ILP over propositional learning techniques such as logistic regression are that it can utilize data from relational databases with many tables, discover rules that are based on logic easily understood by humans and computers, and finally, it can generate rules that can provide meaningful insight about predictive indicators that distinguish the negative examples from positive examples.^[12]^ The objective of this study was to assess rules for prediction of thyroid cancer recurrence from our institutional database using inductive logic programming.

## 2 Methods

Among the patients who visited the Thyroid Cancer Clinic at Yonsei University College of Medicine between January, 2009 and June, 2010 as a result of receiving a diagnosis of WDTC, 797 patients who underwent bilateral total thyroidectomy with central compartment lymph node dissection and radioiodine treatment, and were followed up for more than 5 years, were included in this study. Of the 797 patients, 12 patients with missing recurrence data were excluded. This study was carried out in accordance with the principles laid out in the World Medical Association's Declaration of Helsinki, Good Clinical Practice, and associated Korean regulations. This study was approved by the Institutional Review Board of Gangnam Severance Hospital, Yonsei University College of Medicine, Korea (IRB number 3-2018-0079). As data were obtained retrospectively, informed consent is not mandatory for retrospective studies in Korea, the institutional review board waived the need for informed consent.

Clinical parameters (age, gender, and body mass index [BMI]), pathological information (cancer size, extrathyroidal extension, multiplicity, central compartment lymph node metastasis, lateral neck lymph node metastasis, and thyroiditis), genetic information (BRAF gene mutation), laboratory parameters (fT4, TSH, thyroglobulin, anti-TPO antibody, anti-thyroglobulin antibody before and after surgery, thyroglobulin levels 1, 2, 3, 4, and 5 years after surgery), and the frequency of radioiodine ablation therapy, radioablation doses, and recurrence were collected for analysis (Table S1, Supplemental Digital Content). Recurrence was diagnosed on the basis of whether it was confirmed through pathological and structural information. Finally, the DELMIA Process Rules Discovery was used for analysis. Inductive logic programming was used to extract rules that represents algorithms to predict recurrence. To create algorithms which detect recurrence, 624 cases (79.5%) were used, whereas 161 cases (20.5%) were analysed for validation of created rules.

## 3 Results

### 3.1 Creation of rules

Of the total of 785 cases, 624 (79.5%) cases were used for creating rules, whereas 161 cases (20.5%) were used for validation of created models.

Among the 624 cases, there were 43 (6.9%) recurrences, whereas 581 patients (93.1%) were recurrence free (Table 1).

**Table 1:** Data set for modelling of rules.

	No recurrence	Recurrence	Total
Cases (n)	581	42	624
%	93.1	6.9	100

**Figure 1:**
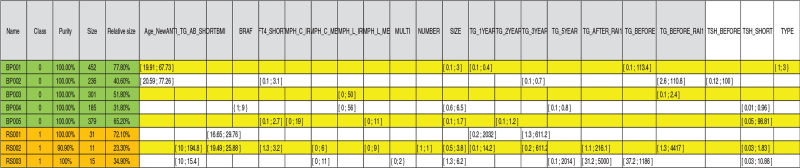
Rules for the prediction of cases with and without recurrence. BP001 to 005 are rules for cases without recurrence; RS001, RS002, and RS003 are rules which predict recur.

In total, 5 rules were identified that could predict the 581 patients without recurrence, whereas 3 rules were identified that predict the 43 cases with recurrence (100%) (Fig. 1).

**Rule 1** predicted that 31 patients had recurrence (72.10%) and represented the sum of the following parameters: BMI (16.65-29.76 kg/m^2^) AND thyroglobulin level at 1 year (0.2-2032 (ng/mL) AND thyroglobulin level at 3 years (1.3-611.2 ng/mL). All patients who met the criteria of rule 1 had recurrences, and there were no patients without recurrence who met the above criteria (purity 100%).

**Rule 2** included 11 patients (23.30%) with recurrence who had the following characteristics: an anti-thyroglobulin antibody level of 10 to 194.8 IU/mL; a BMI of 19.49 to 25.88 kg/m^2^; a free T4 concentration of 1.3 to 3.2 ng/dL; central lymph node metastasis, 0 to 6 in number; lateral lymph node metastasis, 0 to 9 in number; tumor size of 0.5 to 3.8 cm; thyroglobulin at 1-year follow-up of 0.1 to 14.2 ng/mL; thyroglobulin at 3-year follow-up of 0.2 to 611.2 ng/mL; thyroglobulin at 5-year follow-up of 0.1 to 2014 ng/mL; thyroglobulin after radioiodine (RAI) of 1.1 to 216.1 ng/mL; thyroglobulin before RAI of 1.3 to 4417 ng/mL; and a TSH after surgery of 0.02 to 1.8 mcIU/mL3.

**Rule 3** described 15 patients (34.90%) with recurrence and was defined as the sum of the following parameters: anti-thyroglobulin antibody level of 10to 15.4 IU/mL, number of central lymph node metastasis of 0 to 11 regardless of multiplicity, tumor size of 1.3 to 6.2 cm, 5-year thyroglobulin level of 0.1 to 2014 ng/mL, thyroglobulin after RAI of 31.2 to 5000 ng/mL, thyroglobulin level before RAI of 37.2 to 1186 ng/mL, and TSH after surgery of 0.03 to 10.88 mcIU/mL.

A total of 5 rules were identified that described all patients without recurrence.

### 3.2 Validation of created rules

In the validation group, 11 (7.0%) recurrences were observed among the 159 patients (Table 2).

**Table 2:** Data set for validation of created rules.

	No recurrence	Recurrence	Total
Cases	147	14	161
%	91.3	8.7	100

For all created rules, the average prediction success rate was 95.7%. Of the 14 cases with recurrence, only 10 were correctly predicted to be positive for recurrence (success rate 71.4%), whereas 98% of the cases without recurrence were correctly predicted to be negative for recurrence (Tables 3 and 4).

**Table 3:** Validation of created rules to predict recurrence.

	Actual class distribution		
	No recurrence	Recurrence	Total
Prediction			
No recurrence	144	0	144
Recurrence	0	10	10
Abstention	3	4	7
Total	147	14	161

**Table 4:** Validation of success rates.

	Actual class distribution		
	No recurrence	Recurrence	Average
Success rate	98%	71.4%	95.7%
Failure rate	0%	0%	0%
Abstention rate	2%	28.6%	4.3%

## 4 Discussion

In our study, we identified 3 rules that described all patients with recurrence in the model creating group and could correctly predict 71.40% of the recurrences. The most important parameters included in the model were thyroglobulin levels at 1, 2, 3, 4, and 5 years after onset and thyroglobulin levels before and after surgery. Other factors included in the rules were BMI, anti-thyroglobulin antibody, fT4, central and lateral lymph node metastasis, cancer size, and postoperative TSH level.

Although the primary tumor marker for detecting recurrence in cases undergoing total thyroidectomy and radioiodine treatment is the level of thyroid specific thyroglobulin, in patients with thyroglobulin-antibodies, the value can be over- or underestimated, which makes it difficult to detect recurrence.^[11,15,16]^ The serum thyroglobulin level is determined by measuring the residual amount of malignant and normal thyroid tissue, degree of injury to thyroid tissue (including fine needle biopsy, operative resection, and RAI), and levels of thyroid-stimulating hormone.^[17]^

Studies have reported that early postoperative stimulated serum thyroglobulin level is an independent predictor of structural recurrence, and it accurately quantifies the risk of structural disease recurrence. In agreement with these results, our study suggests that early postoperative thyroglobulin could be employed in risk stratification using a serum thyroglobulin level of <2 ng/mL as a cut-off to guide adjuvant therapy and determine the frequency of surveillance in patients with lower early postoperative thyroglobulin.^[18]^

## 5 Conclusions

This study is the first study that attempted to predict thyroid cancer recurrence using machine learning models. Although the prediction rate was relatively high, the clinical meaning and everyday clinical application should be further clarified. Nevertheless, the results of our study show that ILP with validation can be reliably used to help in the identification of novel hypotheses for recurrence in thyroid cancer patients.

## Author contributions

Kim SY made contributions to conception, design of the work, acquisition, analysis, interpretation of data and drafted the work.

Kim YI made contributions to analysis and interpretation of data.

Kim HJ, Chang H, Kim SM, Lee YS made contributions to the conception, design of work and acquisition.

Kwon SS and Shin H made contributions to analysis and interpretation of data.

Chang HS and Park CS substantially revised the manuscript.

All authors have approved the submitted version and have agreed both to be personally accountable for the author's own contributions and to ensure that questions related to the accuracy or integrity of any part of the work.

**Conceptualization:** Soo Young Kim, Hee Jun Kim, Hojin Chang, Seok-Mo Kim, Yong Sang Lee.

**Data curation:** Soo Young Kim, Hee Jun Kim, Hojin Chang, Seok-Mo Kim, Yong Sang Lee.

**Formal analysis:** Soo Young Kim, Young-Il Kim, Soon-Sun Kwon, Hyunjung Shin.

**Methodology:** Soo Young Kim.

**Software:** Young-Il Kim.

**Supervision:** Soon-Sun Kwon, Hyunjung Shin.

**Writing – original draft:** Soo Young Kim.

**Writing – review & editing:** Soo Young Kim, Hang-Seok Chang, Cheong Soo Park.

## Supplementary Material

Supplemental Digital Content
