# Supplementary material for: New approach of prediction of recurrence in thyroid cancer patients using machine learning
Source: Medicine (Baltimore). 2021 Oct 22;100(42):e27493. doi: 10.1097/MD.0000000000027493 (PMC8542129; doi:10.1097/MD.0000000000027493)
Supplement: Supplemental Digital Content [file medi-100-e27493-s001.docx]

Supplemental Table 1: Parameters collected for analysis

| **Abbreviation** | **Parameters** |
| --- | --- |
| Age_New | Age |
| Sex | Female/male |
| BMI | Body mass index |
| Type | Pathologic type (papillary, follicular or both) |
| Size | Cancer size |
| NUMBER | Number of cancer |
| Extrathyroidal extension | Extrathyroidal extension |
| MULTI | Multiplicity |
| LYMPH_C_IRRA | Harvested central lymph nodes |
| LYMPH_C_META | Metastatic central lymph nodes |
| LYMPH_L_IRRA | Harvested lateral neck lymph nodes |
| LYMPH_L_META | Metastatic lateral neck lymph nodes |
| Thyroiditis | Presence of thyroiditis |
| BRAF | BRAF mutation status |
| FT4_BEFORE | fT4 level before surgery |
| TSH_BEFORE | TSH level before surgery |
| TG_BEFORE | Thyroglobulin Ag before surgery |
| ANTI_TPO_AB_BEFORE | Anti-TPO antibody before surgery |
| ANTI_TG_AB_BEFORE | Anti-thyroglobulin antibody before surgery |
| FT4_SHORT | fT4 shortly after surgery (1^st^ visit) |
| TSH_SHORT | TSH level shortly after surgery (1^st^ visit) |
| TG_SHORT | Thyroglobulin Ag shortly after surgery (1^st^ visit) |
| ANTI_TG_AB_SHORT | Anti-thyroglobulin antibody shortly after surgery (1^st^ visit) |
| TG_1YEAR | Thyroglobulin Ag, 1 year postoperative |
| TG_2YEAR | Thyroglobulin Ag, 2 year postoperative |
| TG_3YEAR | Thyroglobulin Ag, 3 year postoperative |
| TG_4YEAR | Thyroglobulin Ag, 4 year postoperative |
| TG_5YEAR | Thyroglobulin Ag, 5 year postoperative |
| TG_BEFORE_RAI1 | Thyroglobulin Ag before radioiodine treatment |
| TG_AFTER_RAI1 | Thyroglobulin Ag after radioiodine treatment |
| After Tg- Before Tg | Thyroglobulin Ag level after radioiodine treatment- Thyroglobulin Ag level before radioiodine treatment |
| BeforeTg/AfterTg | (Thyroglobulin Ag level before radioiodine treatment)/ (Thyroglobulin Ag level after radioiodine treatment) |
